# A Novel Wearable Device for Food Intake and Physical Activity Recognition

**DOI:** 10.3390/s16071067

**Published:** 2016-07-11

**Authors:** Muhammad Farooq, Edward Sazonov

**Affiliations:** Department of Electrical and Computer Engineering, University of Alabama, Tuscaloosa, AL 35401, USA; mfarooq@crimson.ua.edu

**Keywords:** wearable sensor, activity monitoring, food intake monitoring, chewing, support vector machine (SVM), energy intake, energy expenditure, piezoelectric strain sensor

## Abstract

Presence of speech and motion artifacts has been shown to impact the performance of wearable sensor systems used for automatic detection of food intake. This work presents a novel wearable device which can detect food intake even when the user is physically active and/or talking. The device consists of a piezoelectric strain sensor placed on the temporalis muscle, an accelerometer, and a data acquisition module connected to the temple of eyeglasses. Data from 10 participants was collected while they performed activities including quiet sitting, talking, eating while sitting, eating while walking, and walking. Piezoelectric strain sensor and accelerometer signals were divided into non-overlapping epochs of 3 s; four features were computed for each signal. To differentiate between eating and not eating, as well as between sedentary postures and physical activity, two multiclass classification approaches are presented. The first approach used a single classifier with sensor fusion and the second approach used two-stage classification. The best results were achieved when two separate linear support vector machine (SVM) classifiers were trained for food intake and activity detection, and their results were combined using a decision tree (two-stage classification) to determine the final class. This approach resulted in an average F1-score of 99.85% and area under the curve (AUC) of 0.99 for multiclass classification. With its ability to differentiate between food intake and activity level, this device may potentially be used for tracking both energy intake and energy expenditure.

## 1. Introduction

Automatic monitoring of food intake is critical to understand and study the factors that contribute towards the development of obesity and eating disorders [1,2]. Traditional methods for monitoring of ingestive behavior rely on self-reporting techniques, such as food frequency questionnaires [3], 24-h recall [4], and use of mobile devices [5]. These methods suffer from limitations imposed by participant burden, and inaccuracies in self-reporting the data due to reliance on human memory [6,7,8]. In recent years, to alleviate problems associated with self-reporting, several wearable devices have been proposed for automatic detection and monitoring of food intake. Sensor systems used for this purpose can be divided into two groups—sensors placed in the environment and body worn/wearable sensors. Camera-based systems are examples of sensors placed in the environment for monitoring eating episodes [9,10]. In [9], a multistage algorithm was proposed to detect chewing from surveillance videos. The proposed algorithm involved detection of the mouth region and computation of spatiotemporal frequency spectrum of the small perioral region for recognition of chewing movements. A similar system presented in [10], using Active Appearance Models (AAM) for detection of faces in surveillance videos; it used observed variations in AAM parameters for detection of chewing, with an average accuracy of 93%, for 37 participants. Sensors placed in the environment around individuals cause the least burden to participants but suffer from limitations such as the need for specially equipped spaces and restriction on participant’s movements, since they need to be in the field of view of the camera. Additionally, low lighting conditions can also hinder the performance of these systems. 

Body-worn sensors have been proposed to capture different stages of eating, such as bites, chewing and swallowing of food. Several hand/wrist-worn wearable devices—including accelerometers, gyroscopes and smart watches [10,11,12,13]—have been proposed for detection of gestures related to eating. In [11], Dong et al. proposed the use of a wrist-worn device (in the form of a watch) to track wrist movements associated with food intake, and were able to detect and count bites taken during a meal with an accuracy of 86% in cafeteria settings. In [14], a wearable sensor system with five inertial sensors located on the wrists, upper arms, and upper torso was proposed. Some researchers have suggested wearing audio recording devices on the wrists to record sound, and use machine learning and pattern recognition algorithms to detect eating episodes based on the recordings [13,15]. Wrist-worn sensors are the most natural option, but they are relatively inaccurate compared to the other food intake monitoring systems.

The second stage of eating involves chewing and can be monitored via chewing sounds [16,17,18], EMG and force sensors [19,20,21], or capturing jaw vibrations during chewing using strain sensors [22,23,24,25]. In [18], use of a conduction microphone was suggested for capturing chewing sounds. An acoustic based approach for detection of chewing suffers from the presence of environmental acoustic noise and, therefore, requires the use of additional reference microphones to eliminate environmental noise [18,26]. Another possibility is to measure the deformation in the ear canal walls using proximity sensors due to chewing activities during food intake [27]. In [27], a wearable sensor system in the form of an earpiece was proposed, which included three infrared proximity sensors placed orthogonally with respect to each other to allow for a wider coverage of the ear canal. Participant dependent models detect the presence of food intake with an accuracy of 93% in a laboratory setting. In [28], the use of a single axis accelerometer placed on the temporalis was proposed to monitor chewing during eating episodes in laboratory experiments. The characteristic jaw movements produced during chewing can also be captured by using a piezoelectric strain sensor [23]. A three module sensor system (hand gesture sensor, accelerometer, and piezoelectric strain sensor below the ear) was used by 12 participants to monitoring food intake in free-living conditions for 24 h [22,25,29]. The system was able to detection presence of chewing with an average accuracy of 89% in unrestricted free living conditions. 

Swallowing involves the passage of a bolus of food or liquid from the mouth to the stomach and involves contraction and relaxation of muscles of the tongue (oral preparation), pharynx (the pharyngeal) and esophagus (esophageal phase) [30]. Wearable sensors—such as microphones placed in the ear (to capture swallowing sounds) or on the throat, or surface electromyography—have been proposed to monitor these muscle contractions and relaxations for detection of food intake. In [31], researchers used two microphones for capturing swallowing sounds and environmental noise. Other researchers have proposed similar systems [32,33,34,35] where miniature microphones were placed on the laryngopharynx for automatically differentiating between swallowing sounds related to food intake and other activities. Acoustic based swallowing detection systems suffer from environmental noise and presence of surrounding speech/talking. Another possibility is to use bio-impedance measurement (such as Electromyography (EMG) or Electroglottography (EGG)) at larynx level for detection of swallowing related to food intake [36,37]. A piezoelectric strain sensor placed against the throat is subjected to physical strain during muscle contraction and relaxation caused by swallows [38,39]. These systems are virtually insensitive to the environmental noise. However, they are not able to reliably distinguish swallows from the head and neck movements and, therefore, their use in free-living conditions is limited [34]. 

Most of the systems presented in the literature were tested either in laboratory conditions or assume food intake episodes in sedentary postures. People can eat food while in a sedentary posture or while performing low to moderate intensity activities (in the range of 3–6 metabolic equivalents (MET)), such as walking [22,40]. Several accelerometer-based physical activity monitoring systems have been proposed in the literature to recognize physical activities of different intensities, such as sitting, standing, walking, etc. [41,42]. To the best of our knowledge, no system has been tested for detection of food intake when participants were physically active. The purpose of this work is to present a new novel wearable sensor system for automatic, accurate and objective monitoring of ingestive behavior that reliably recognizes food intake in physically active users, which makes it potentially suitable for detection of snacking “on the go.” The proposed system also has the ability to differentiate between the states of low physical activity (sedentary state) and moderate physical activity such as walking. The device is integrated into eyeglasses with a piezoelectric strain sensor placed on the temporalis muscle and an accelerometer placed on a temple of the eyeglasses. Food intake detection is performed using features from the piezoelectric strain sensor, and the intensity of physical activity is detected using an onboard accelerometer. Two multiclass classification approaches are proposed to differentiate between different classes of activities. The wearable device presented in this work is non-invasive, unobtrusive, and potentially socially acceptable. The device has the potential ability to track both energy intake and energy expenditure patterns, which could be used in developing strategies for understanding and tackling obesity. 

## 2. Materials and Methods 

### 2.1. Data Collection Protocol

A total of 10 volunteers were recruited for this study (8 male and 2 female, average age 29.03 ± 12.20 years, range 19–41 years, average body mass index (BMI) 27.87 ± 5.51 kg/m^2^, range 20.5 to 41.7). Participants did not suffer from any medical conditions which would impact their chewing. Before the start of the experiment, each participant signed an informed consent form. University of Alabama’s Institutional Review Board (IRB Protocol # 12-011-ME-R3) approved the study. For training and validation of classification models, an accurate reference (gold standard) is required, which at the moment, is not possible to attain in unrestricted free-living conditions. Therefore, the experiments were performed in a laboratory, where it was possible to observe participants closely and develop an accurate reference. During a single visit to the laboratory, participants followed a protocol which started with an initial five minute quiet rest, during which participants were asked to use their phone or computer without talking while sitting. Next, the participants consumed a slice of cheese pizza while sitting comfortably in a chair. They were allowed to talk during the meal. The food intake was followed by five minutes of talking or reading out loud. Next, participants were asked to eat a granola bar while walking on a treadmill at 3 mph. The final activity was walking on the treadmill for 5 min at 3 mph, where they were not asked to eat anything. 

Activities performed by the participants were representative of the activities performed in daily free-living conditions. Pizza represented a food item that may be consumed in a meal or a snack. Granola bar represented snacks that may be consumed on the go. A speed of 3 mph was chosen for ambulation because the normal walking speed varies from 2.8 mph to 3.37 mph depending on the age of individuals [43]. Throughout the experiment, there were no restrictions on participant’s body movements (including head movements), and they were allowed to talk throughout the experiment, even during eating. 

### 2.2. Sensor System and Annotation 

**Figure 1:**
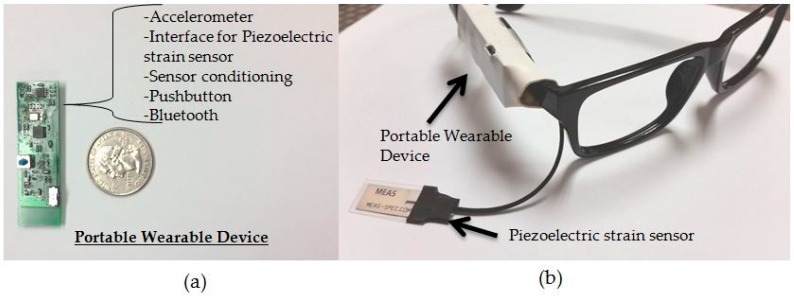
(**a**) Portable wearable device for monitoring of food intake and level of physical activity. The data acquisition module also has an accelerometer and Bluetooth; (**b**) Eyeglasses with a piezoelectric sensor and data acquisition device connected to the temple of glasses.

**Figure 2:**
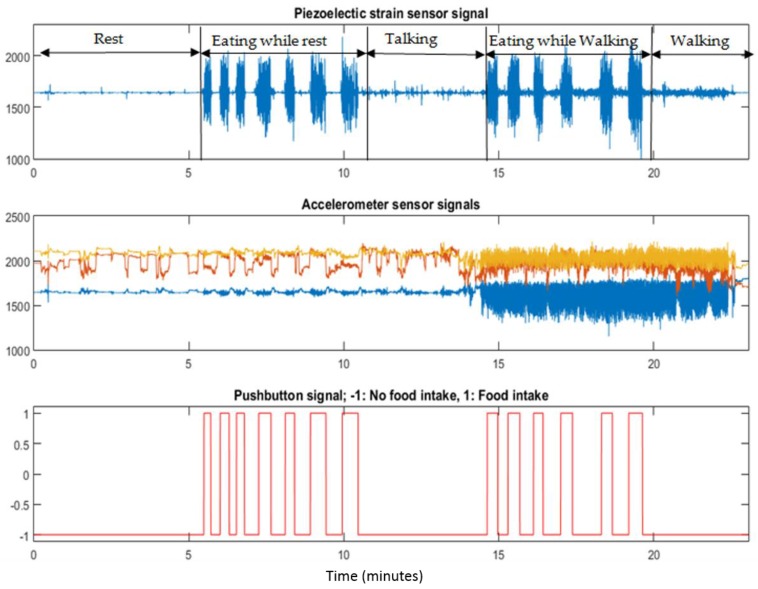
Sensor signals collected during the experiment. Piezoelectric sensor signals (first row) and accelerometer signals (second row) are used to differentiate between eating and physical activities. Eating episodes were marked by participants using a pushbutton (third row).

The wearable device proposed in this work combined the data collection, signal conditioning, and wireless data transmission into a single module that was connected to the temple of the eyeglasses. Jaw movements during chewing were captured by placing a commercially available piezoelectric film sensor (LDT0-028K from Measurement Specialties Inc., Hampton, VA, USA) on the temporalis muscle using medical tape. Temporalis muscle is part of the muscles that controls the jaw movements during chewing [44]. An ultra-low power operational amplifier (TLV-2452, Texas Instruments, Dallas, TX, USA) with an input impedance of 1 GΩ was used to buffer the high impedance of the piezoelectric sensor. Sensor signals were sampled at 1000 Hz by a microprocessor (MSP430F2418, Texas Instruments, Dallas, TX, USA) by a 12-bit analog to digital converter (ADC). Body acceleration was recorded with a low-power three axis accelerometer (ADXL335 from Analog Devices, Norwood, MA, USA) and sampled at 100 Hz. Participants used a pushbutton to mark chewing sequences (sampled at 100 Hz) and to define boundaries of different activities. Collected sensor data were sent via an RN-42 Bluetooth module with serial profile to an Android Smartphone for storage and further offline processing in MATLAB (Mathworks Inc., Natick, MA, USA). Figure 1 shows the piezoelectric strain sensor and the data collection acquisition module connected to the temple of the glasses. Figure 2 shows example signals of the piezoelectric strain sensor and accelerometer sensor during different activities.

### 2.3. Signal Processing and Feature Computation

Chewing frequency is in the range of 0.94 to 2.17 Hz [45], therefore, piezoelectric sensor signals were passed through a low-pass filter with a cut-off frequency of 3.0 Hz. For feature computation, both piezoelectric sensor and accelerometer signals (net acceleration) were divided into non-overlapping 3-s epochs. Selected epoch size ensures that even for the lower bound of chewing frequency, an epoch will contain multiple chews. Close observation of Figure 2 suggests that, irrespective of the activity level of the participants (sitting or walking), for the piezoelectric strain sensor, eating episodes have higher energy compared to non-eating episodes. A similar trend can be seen in the accelerometer signal, where walking registers higher energy (based on amplitude variations) compared to being physically sedentary. Features listed in Table 1 were computed to represent the sensor signals for classification. 

**Figure 3:**
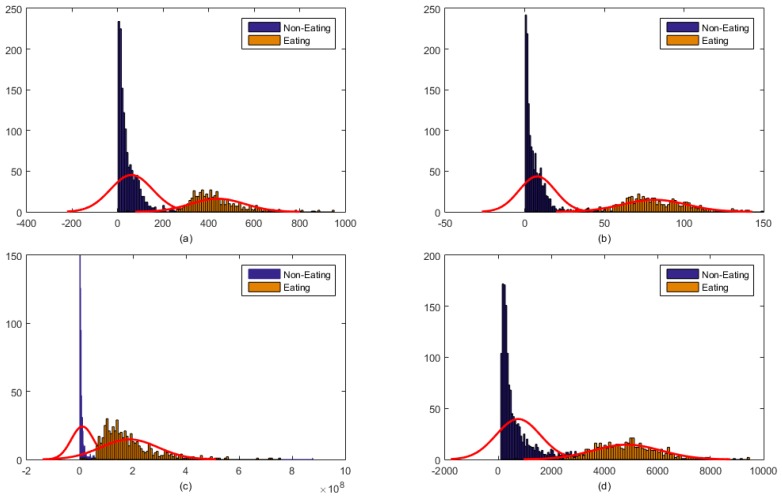
Histogram showing the distribution of piezoelectric strain sensor signal features: (**a**) Range of values (**b**) Standard deviation (**c**) Energy (**d**) Waveform length. The feature distribution shows that these features can easily provide information for separation of food intake from non-intake.

**Figure 4:**
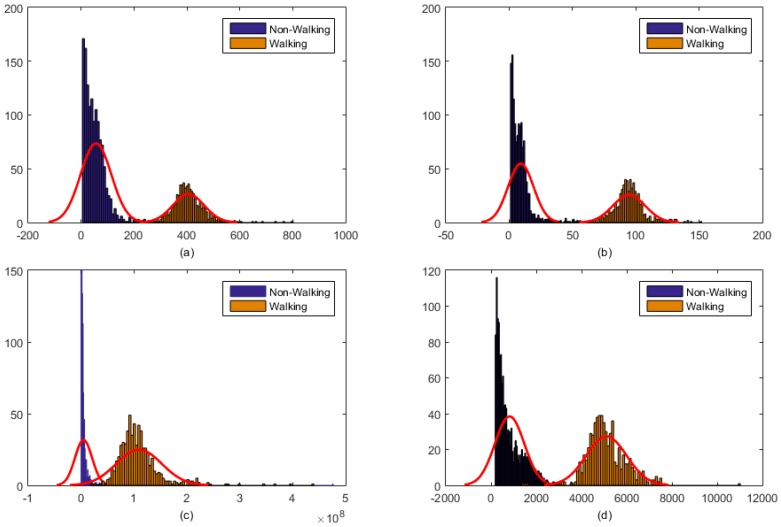
Distribution of accelerometer sensor signal features: (**a**) Range of values (**b**) Standard deviation (**c**) Energy (**d**) Waveform length. Feature distribution shows that these features can easily provide information for separation of walking from the non-walking activity.

For the *i^th^* epoch, the piezoelectric strain sensor feature vector was represented by *f_i,chew_* and the feature vector for the accelerometer signal was represented by *f_i,Acc_*. A pushbutton was used to define the class label for a given epoch. Figure 3 shows the distribution of piezoelectric strain sensor features for two classes: eating and not eating, irrespective of the activity level. Similarly, Figure 4 shows the distribution of features computed from accelerometer signals for two classes: epochs with physical activity (walking) and without physical activity (sedentary), irrespective of whether the participant was eating or not eating. From the given feature distributions, it is clear that these classes are easily separable using the respective feature vectors. 

### 2.4. Multiclass Classification

Each participant performed five different activities which were: sitting quietly, eating while sitting, speaking while resting, eating while walking, and walking. Analysis of the piezoelectric strain sensor features for the rest (sitting quietly) and speaking while resting shows that the distributions of the features for these two classes are similar. Therefore, sitting quietly and sitting while talking were combined into a single class called sedentary. Thus, data were reduced to four classes: sedentary, eating while sitting, eating while walking, and walking. In this work, a linear support vector machine (SVM) was used for classification. All classifiers were trained using the Classification Learner App in MATLAB 2015 (Mathworks Inc., Natick, MA, USA). Two different approaches were tested for multiclass classification using features from both sensors i.e., single multiclass classification and multistage classification. 

#### 2.4.1. Single Multiclass Linear SVM with Sensor Fusion 

In this approach, for a given epoch, piezoelectric and accelerometer features were combined into a single feature vector, i.e., *f_i_* = {*f_i,chew_*,*f_i,Acc_*}. A human expert assigned multiclass labels to different activities based on the activity boundaries marked with the pushbutton by the participants. Epochs were assigned labels for four classes based on the reassigned human expert labels as *C_i_ = {1: Eating while sitting, 2: Sedentary, 3: Eating while walking, 4: Walking}.* A multiclass linear SVM classifier was trained using a one-vs.-all strategy.

#### 2.4.2. Multi-Stage Classification: Linear SVM + Decision Tree 

This approach used two stage classification where the first stage used two different classifiers. The first classifier detected food intake solely based on the piezoelectric sensor signals. The second classifier recognized physical activity to differentiate between walking and sedentary classes based on accelerometer signals. Both classifiers were binary linear SVM models. The food intake detection classifier was trained with piezoelectric strain sensor features i.e., *f_i,chew_*, and predicts the class label *C_i,chew_* = {−1: No-Food Intake, 1: Food Intake}. The activity recognition classifier was trained with accelerometer features (*f_i,Acc_*) and class labels are predicted as *C_i,Acc_* = {−1: No-Walking, 1: Walking}. The second stage classification used a simple decision tree to estimate the final class label *C_i_.* A decision tree implemented empirical rules given in Table 2. 

Each classifier was trained with leave-one-out cross-validation scheme, where data from nine participants were used for training the classifier, and the remaining participant was used for evaluating the performance of the classifier. This process was repeated 10 times, so that each participant was used once for validation. For each class of activities, the F1-score was computed, which is the weighted average of precision and recall. The F1-score was computed as follows:

F1-score = 2 × Precision × Recall/(Precision + Recall)
(1)

Precision = TP/(TP+FP)
(2)

Recall = TP/(TP+FN)
(3)

where TP, FP, and FN denote true positives, false positives, and false negatives for each class, respectively. Additionally, data from all validation participants were combined to plot the Receiver Operation Characteristic (ROC) curve and compute Area Under the Curve (AUC) for each class. 

## 3. Results

The dataset used in the study included four different classes with 322 epochs of eating while sitting, 1155 epochs of sedentary, 271 epochs of eating while walking and 437 epochs of walking. Table 3 presents the confusion matrix for classification results of the single multiclass classifier. Results show that this classification approach was able to differentiate between four classes, with an average F1-score of 95.67% and average precision and recall of 95.42% and 95.93%, respectively. The lowest F1-score, of 93.85%, was achieved for classification of walking, where 17 walking epochs were misclassified as eating while walking. Table 4 shows the confusion matrix for the two-stage classification approach. In this case, the classifier was able to achieve average F1-score of 99.85% for all classes with average precision and recall of 99.89% and 99.82%, respectively. 

**Figure 5:**
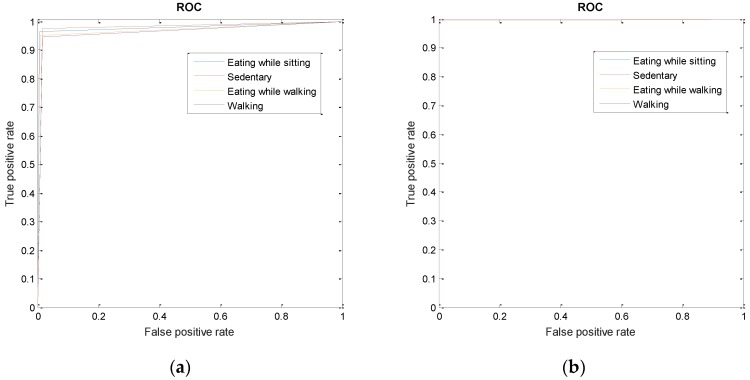
Receiver Operation Characteristics (ROC) Curves for two classification approaches. (**a**) ROC curves for different classes when single linear SVM model is trained; (**b**) ROC curve for two-stage classification. The first stage uses two linear SVM models for detection of food intake and walking, after which a simple decision tree is used to predict the final output class.

Figure 5 shows the Receiver Operation Characteristics (ROC) curves for each class for both classifications approaches (Figure 5a for the single multiclass classifier; Figure 5b for the two-stage classification procedure). ROC curves show that the two-stage classification approach produced better results for all classes, compared to the single multiclass classifier. Table 5 also lists the Area under the Curve (AUC) for each class for both classification approaches. For single multiclass classifier, the average AUC for all classes was 0.97. For two-stage classification; the classifier was able to achieve an average AUC of 0.99. 

## 4. Discussion

It is critical to develop new techniques for automatic, objective, and accurate monitoring of food intake to overcome the limitation posed by current methods, which rely on self-reporting of food intake. Most of the systems proposed in the literature either have not been tested in free-living conditions or do not include activities which replicate such conditions. The majority of previous studies consider food intake when participants are at rest or sedentary [23,27,28,36,46,47]. Although some people may consume food while performing moderate intensity activities (in the range of 3−6 metabolic equivalents (MET) [22,40]), most of the systems reported do not consider food intake while the participants are moving (e.g., walking). A possible reason is that the presence of motion artifacts may impact the sensor signals and hence impact the performance of the classification algorithm. This work presents a study of a novel wearable sensor system which can detect the presence of food intake both when participants are at rest/sedentary or in motion (physically active). This wearable device can also differentiate between the being in a sedentary or physically active state. A piezoelectric strain sensor was used for the detection of chewing during food intake and an accelerometer was used for detection of whether the participant is sedentary or in motion. 

This work presents two approaches for multiclass classification. Sensor fusion combined with single multiclass classifier resulted in average F1-score of 95.67% for four classes. For this approach, misclassification occurs among all classes (Table 3). For the sedentary class, a total of 27 misclassifications occurred (only 2.35% misclassification for sedentary), where 11 epochs were misclassified as eating while sitting and 16 epochs are misclassified as walking. The highest misclassification rate occurred for eating while walking (5.54%), followed by walking (5.26%). The highest number of misclassification occured between eating while walking and walking classes, which was expected, since the accelerometer signals have similar amplitude for both of these classes (see Figure 2). 

The two-stage classification approach reduced the misclassifications. Two separate classifiers were used for food intake detection and walking, and results of both classifiers were combined using a simple decision tree to achieve multiclass classification. The final classifier achieved an average F1-score of 99.85% for all classes with 10-fold cross-validation scheme. Figure 5b depicts the ROC for each class, with an average AUC of about 0.99 for all classes. This approach resulted in only two misclassifications out of 2185 epochs. Two eating while walking epochs were misclassified as walking.

The results of this work show that both single multiclass and two-stage multiclass classifiers performed with a satisfactory level of robustness. Models developed in the study were based on the whole population (participant-independent) and, therefore, no participant-specific calibration was required. This is critical, as it ensures the usage of the device in the general population. 

Compared to the current state of the art for automatic detection and monitoring of eating behavior, the device presented in this work performed accurate food intake detection in more challenging test conditions. Several wearable solutions have been presented for monitoring of food intake where the accuracy of food intake detection ranges from 80% to 96% [22,27,36,38,39]. Most of these systems have not been tested in the presence of physical activity or other motion artifacts, such as head movements. For these solutions to be employed in real life conditions, it is critical for them to be robust in the presence of such conditions. The device and algorithms presented in this work are robust and not impacted by the presence of walking, speech and head movements. The device also has the ability to recognize whether the participants are sedentary or physically active (walking). Tested in challenging conditions, the proposed device also proved more accurate compared to the state of the art. 

In the proposed device, the data collection module is connected to the temple of eyeglasses, which reduces the number of sensors a participant has to wear compared to a multi-sensor system previously presented [22]. This helps in potentially reducing user burden and increasing user compliance. Eyeglasses make the design potentially non-invasive, unobtrusive, and socially acceptable. The device can be further miniaturized and embedded into a headband or hat/cap for people who do not wear eyeglasses. In future, both the feature computation as well as the classification can be implemented either on the device or on a phone to avoid offline processing, and provide feedback to the user in real time. Further, a camera can be integrated into the current design of the eyeglasses which may be triggered to take images of the food whenever the system detects food intake. Computer vision algorithms can be used for recognizing food items from these images [48]. 

A limitation of the present study is that the device’s ability to detect intake of liquids/beverages was not tested, as the main focus was on the detection of solid food. Research suggests that even during continuous liquids intake (sips), characteristic jaw movements similar to chewing are present and hence can be used for detection of liquid intake [23]. Detection of liquids will be explored in future research. Another limitation is that the current study was performed in a laboratory setting because of the need for accurate reference/gold standard. The current study was performed to establish the feasibility of the sensor system and further studies free-living studies will be conducted to test the system in more realistic settings. During the design of the experiment, activities such as walking, speech, head movements, etc. were included to replicate some of the activities performed by people in their daily living. However, the included activities were not full representative of the activities performed by individuals in real world settings, therefore, unrestricted free living studies are required to test the system in real life conditions. A significant decrease of performance is not anticipated in the unrestricted free living conditions, as some of the complex activities were included in the protocol. The results are presented for a relatively small cohort of 10 individuals. Further, long-term studies with larger cohorts will be conducted to test the system. The variety of foods included in the study were limited and needs to be further extended for evaluation of the system on wide range of food with different physical properties. The system utilized a pushbutton by the participants to establish a gold standard for algorithm development. There is a possibility that using a pushbutton and the sensor system might change the way participants are eating, however, even with this potential change, the system is able to accurately track eating. Other methods such as video observations annotated be human raters can be used for the development of a gold standard, which will allow for participant independent labelling of the data. However video-based annotation is dependent on the quality and resolution of the videos. Finally, the piezoelectric sensor was placed on the temporalis muscle using medical tape which might limit the long term use and obtrusiveness of the system. While no sensor detachment was observed during the experiments, future implementations may include integration of the sensor into the temple of the eyeglasses to improve user comfort and practicality of the system.

For a healthy lifestyle, it is critical to maintain a balance between energy intake and energy expenditure patterns [49]. Energy expenditure is associated with the level of physical activity performed by individuals. Accelerometer based physical monitoring systems proposed in the literature are able to differentiate between different activities such as walking, sitting, standing, walking upstairs, walking downstairs [41,42]. Each of these physical activities results in a different level of energy expenditure. The proposed system in the current form can only differentiate between low (sitting/sedentary) and medium to high level of physical activity (walking). There is a potential that the system can further be developed to recognize a wider range of physical activities. We believe that with these added capabilities, the proposed system will be able to track both energy intake and energy expenditure patterns using a single device and can be used to provide valuable feedback to the users for maintaining a healthy lifestyle.

## 5. Conclusions 

This paper presents a wearable device for automatic detection for food intake in the presence of physical activity and motion artifacts. The device is connected to the temple of glasses and combines a piezoelectric strain sensor, accelerometer, and Bluetooth connectivity. Two approaches for multiclass classification are proposed for detection of food intake in the presence of motion artifacts originating from physical activity, speech, and body movement. The proposed device detected periods of food intake with a high average F1-score of 99.85%. With further development, the device has the potential ability to track both energy intake and energy expenditure, and monitor energy balance of individuals. 

## Figures and Tables

**Table 1:** Feature sets computed from both piezoelectric and accelerometer sensor epochs.

No.	Feature	Description *
**1**	Range of values	Rng(x(i)) = Max(x(i)) − Min(x(i))
**2**	Standard deviation	STD(x(i)) = $\sqrt{\sum (x(i)-\bar{x\left(i\right)})/\left(N-1\right)}$
**3**	Energy	Eng(x(i)) = $\sum_{n=1}^{N}x{\left(n\right)}^{2}$
**4**	Waveform length	WL(x(i)) = $\sum_{n=1}^{N-1}\left|x{\left(i\right)}_{n+1}-x{\left(i\right)}_{n}\right|$

* *i* represents epoch number, *n* is the *n^th^* sample in the *i^th^* epoch; *N* *= L* × *f_s_*; *N* = number of samples, *L* = 3; duration of an epoch in second and *f_s_* = 1000; sampling frequency.

**Table 2:** Decision tree rules for determining the final class label from the two-stage classifier.

***If***	***C_i,chew_***		***C_i,Acc_***		***C_i_***
	1		−1		*1: Eating while sitting*
	−1	**and**	−1	***Then***	*2: Sedentary*
	1		1		*3: Eating while walking*
	−1		1		*4: Walking*

**Table 3:** Confusion Matrix for single multiclass linear SVM classifier. Precision, Recall, and F1-score are also listed for each class of activities.

	Eating + Sitting	Sedentary	Eating + Walking	Walking	Recall	F1-Score
**Eating + Sitting**	310	9	3	0	96.58%	96.58%
**Sedentary**	11	1128	0	16	97.66%	98.09%
**Eating + Walking**	0	0	256	15	95.20%	94.16%
**Walking**	0	6	17	414	94.28%	93.85%
**Precision**	96.58%	98.52%	93.14%	93.42%	Mean:	95.67%

**Table 4:** Confusion Matrix for multi-class classification when two stage classification is used. Precision, Recall and F1-score are also listed for each class/categories of activities.

	Eating + Sitting	Sedentary	Eating + Walking	Walking	Recall	F1-Score
**Eating + Sitting**	322	0	0	0	100.00%	100.00%
**Sedentary**	0	1155	0	0	100.00%	100.00%
**Eating + Walking**	0	0	269	2	99.26%	99.63%
**Walking**	0	0	0	437	100.00%	99.77%
**Precision**	100.00%	100.00%	100.00%	99.54%	Mean:	99.85%

**Table 5:** The area under the Curve (AUC) for each class. Mean AUC for each classifier was computed as the average of AUCs for all classes (Mean column).

Classifier	Eating + Sitting	Sedentary	Eating + Walking	Walking	Mean
**Signle multiclass SVM**	0.98	0.98	0.97	0.96	0.97
**Two-stage classification**	1	1	0.99	0.99	0.99

## Abbreviations

The following abbreviations are used in this manuscript:

BMI	Body Mass Index
TP	True Positive
FP	False Positive
FN	False Negative
FP	False Positive
*i*	*i^th^* epoch number
*x(i)*	epoch of sensor signals
*N*	Total number of samples in an epoch
*f_i,chew_*	Feature vector for piezoelectric strain sensor
*f_i,Acc_*	Feature vector for Accelerometer signal
*f_i_*	Feature vector for combined feature vector (sensor fusion)
*L*	length of the epoch in seconds
